# The plug-based nanovolume Microcapillary Protein Crystallization System (MPCS)

**DOI:** 10.1107/S0907444908028060

**Published:** 2008-10-18

**Authors:** Cory J. Gerdts, Mark Elliott, Scott Lovell, Mark B. Mixon, Alberto J. Napuli, Bart L. Staker, Peter Nollert, Lance Stewart

**Affiliations:** a Accelerated Technologies Center for Gene to 3D Structure, USA; b deCODE biostructures Inc., 7869 NE Day Road West, Bainbridge Island, WA 98110, USA; c Emerald BioSystems Inc., 7869 NE Day Road West, Bainbridge Island, WA 98110, USA; d Seattle Structural Genomics Center for Infectious Disease, USA; e Department of Biochemistry, University of Washington, Seattle, WA 98195, USA

**Keywords:** protein crystallization, Microcapillary Protein Crystallization System

## Abstract

The Microcapillary Protein Crystallization System (MPCS) embodies a new semi-automated plug-based crystallization technology which enables nanolitre-volume screening of crystallization conditions in a plasticware format that allows crystals to be easily removed for traditional cryoprotection and X-ray diffraction data collection. Protein crystals grown in these plastic devices can be directly subjected to *in situ* X-ray diffraction studies. The MPCS integrates the formulation of crystallization cocktails with the preparation of the crystallization experiments. Within microfluidic Teflon tubing or the microfluidic circuitry of a plastic CrystalCard, ∼10–20 nl volume droplets are generated, each representing a microbatch-style crystallization experiment with a different chemical composition. The entire protein sample is utilized in crystallization experiments. Sparse-matrix screening and chemical gradient screening can be combined in one com­prehensive ‘hybrid’ crystallization trial. The technology lends itself well to optimization by high-granularity gradient screening using optimization reagents such as precipitation agents, ligands or cryoprotectants.

## Abstract 2

The Microcapillary Protein Crystallization System (MPCS) is a new protein-crystallization technology used to generate nanolitre-sized crystallization experiments for crystal screening and optimization. Using the MPCS, diffraction-ready crystals were grown in the plastic MPCS CrystalCard and were used to solve the structure of methionine-*R*-sulfoxide reductase.

## 1. Introduction

The field of structural biology is generating technologies that increase throughput and efficiency each year. Such advances have initiated a goal to progress from gene to three-dimensional structure in 3 d. In an effort to improve efficiency, one may want to minimize the volume of protein required such that sufficient material for crystallization screening and optimization can be obtained from cell-free synthesis. With the ‘three-day’ structure goal in mind, the Accelerated Technologies Center for Gene to 3D Structure (ATCG3D) is engaged in the development of several technologies to increase efficiency in the gene-to-structure pipeline, including synthetic gene design (Gene Composer; http://www.genecomposer.net/), protein crystal imaging (DETECT-X; Emerald BioSystems) and microfluidic nanovolume crystallization (The Microcapillary Protein Crystallization System; MPCS). Here, we describe the first working prototype of the MPCS, which consists of a microsyringe pumping system, the pumping system’s associated software (*MicroPlugger*) and plastic CrystalCards that house the ‘3+1 mixer’ (where reagents are combined) and microfluidic channel (where experiments are stored). The CrystalCards are manufactured with materials that enable a balance between X-ray transmission, optical clarity, moldability, chemical resistance and surface energy. This system produces diffraction-ready crystals by allowing crystal extraction from the CrystalCard or *in situ* X-ray diffraction and structure solution using diffraction data obtained from samples within the CrystalCard.

## 2. Background

**Figure 1:**
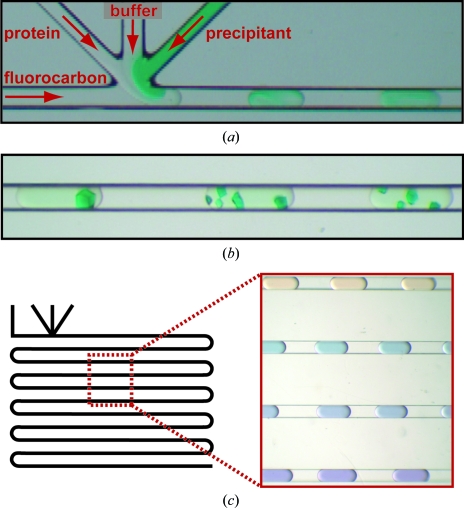
Plug-based protein crystallization using the MPCS. (*a*) A microphotograph of crystallization trials in plugs being formed in the CrystalCard. (*b*) Dyed protein crystals in the MPCS CrystalCard. (*c*) A schematic drawing (left) of a channel containing a smooth pH gradient (right) generated using a low-pH buffer, a high-pH buffer and a universal pH indicator.

The underlying technology used to perform microfluidic nanovolume crystallization discussed here was developed at the University of Chicago (in the ATCG3D-collaborating Ismagilov laboratory). Aqueous droplets, here called plugs, form spontaneously by combining microfluidic aqueous streams with an immiscible and biologically inert fluorocarbon solution (Song *et al.*, 2003 ▶; Tice *et al.*, 2003 ▶, 2004 ▶). Three separate aqueous streams of protein, buffer and precipitant flow simultaneously into a single channel, where a controlled flow of immiscible and biologically inert fluorocarbon breaks the combined aqueous streams into 10–20 nl microbatch-style crystallization experiments (Fig. 1 ▶ *a*; Zheng *et al.*, 2003 ▶; Zheng, Tice, Roach *et al.*, 2004 ▶). The region where the streams converge is called the ‘3+1 mixer’. The 10–20 nl crystallization plugs formed at the ‘3+1 mixer’ are incubated and monitored for crystallization (Fig. 1 ▶ *b*). By controlling the flow rates of the aqueous streams, very fine concentration gradients are generated over a series of plugs (Fig. 1 ▶ *c*), allowing the crystallographer to carefully probe crystallization phase space for crystal optimization. Plug-based crystallization permits sparse-matrix screening (Zheng & Ismagilov, 2005 ▶; Zheng, Tice & Ismagilov, 2004 ▶) using a pre-formed cartridge of plugs, the formation of fine concentration gradients (Zheng *et al.*, 2003 ▶) *via* on-chip formulation of plug composition and a combination of sparse-matrix and gradient screening termed the ‘hybrid method’ (Li *et al.*, 2006 ▶), the latter of which enables comprehensive screening of crystallization phase space in one single crystallization trial. Plug-based microfluidic crystallization has also been used to probe scientific questions regarding protein-crystal nucleation and growth (Chen *et al.*, 2005 ▶; Zheng *et al.*, 2005 ▶; Gerdts *et al.*, 2006 ▶) and to generate crystals for novel protein structures (Gerdts *et al.*, 2006 ▶).

## 3. The MPCS

**Figure 2:**
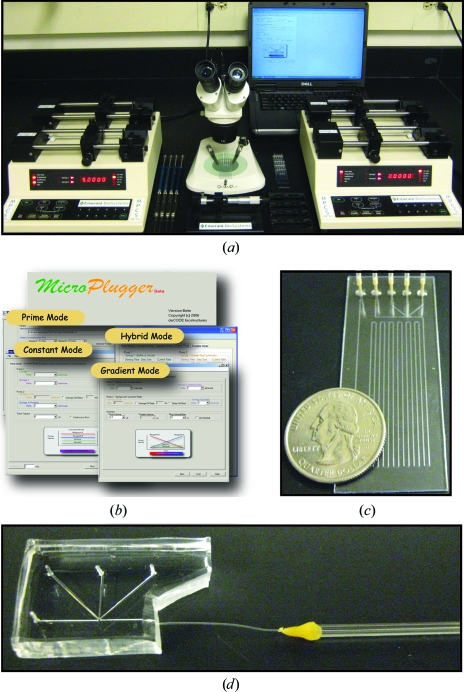
MPCS components. (*a*) A picture of the components of the MPCS system. (*b*) Screenshots of the operational modes of the *MicroPlugger* software. (*c*) A picture of the MPCS CrystalCard filled with dyed protein crystals. (*d*) A picture of the PDMS/Teflon CrystalCard typically used for the hybrid method.

As a technology development center, ATCG3D has developed the Microcapillary Protein Crystallization System (MPCS) in order to make plug-based protein-crystallization technology available to the scientific community. The MPCS instrumentation is composed of three essential parts (Fig. 2 ▶): (i) a pumping system, (ii) the *MicroPlugger* control software and (iii) the CrystalCard. The pumping system is a four-channel syringe-pump system that delivers precise and continuous flows to the MPCS CrystalCard. This system allows independent control over each channel and can generate extremely fine gradients. Pumps are controlled by the *MicroPlugger* pump-control software (Fig. 2 ▶ *b*). The *MicroPlugger* software allows the user to control the pump system in four different operational modes: Prime Mode (for priming the lines), Constant Mode (for constant-flow experiments), Gradient Mode (for generation of fine gradients) and Hybrid Mode (for sparse-matrix screening using the hybrid method). These operational modes allow the user to perform all MPCS experiment styles. The third essential component is the CrystalCard (Figs. 2 ▶ *c* and 2 ▶ *d*), which is the ‘microfluidic chip’ where the aqueous and fluorous solutions come together to spontaneously form the 10–20 nl plugs at the 3+1 mixer. The CrystalCard was designed with a hydrophobic surface that supports the formation of aqueous plugs and contains a long microfluidic capillary for storage and inspection. Access to diffraction-ready crystals is gained *via* physical crystal extraction from the CrystalCard or by *in situ* X-ray diffraction. There are two versions of the MPCS CrystalCard: a plastic CrystalCard and a PDMS/Teflon CrystalCard. The plastic CrystalCard (Fig. 2 ▶ *c*) is used primarily for fine-gradient screening and the PDMS/Teflon CrystalCard (Fig. 2 ▶ *d*) is typically used for hybrid screening and detergent-based membrane-protein formulations. (The PDMS/Teflon CrystalCard has the necessary connection schemes and surface properties to be compatible with hybrid screening and detergent-based crystallizations.)

Using the pumping system, software and CrystalCards, the MPCS generates hundreds of distinct crystallization experiments within a few minutes, free from cross-contamination, using only ∼5 µl protein solution. The MPCS does not waste any precious protein, because there is no dead volume in the system: the protein sample is aspirated directly into a Teflon needle and then displaced in its entirety into the CrystalCard. Additional features of the MPCS include the ability to perform microfluidic seeding (Gerdts *et al.*, 2006 ▶) and the control of evaporation by controlling the humidity of the CrystalCard’s storage container (CrystalCards stored in a humidified container remain unchanged for at least two months).

### 3.1. MPCS experiment styles

Plugs formed using the MPCS are microbatch-style crystallization experiments in which protein and crystallizing agent are combined and incubated while surrounded by an oil (fluoro­carbon, FC40; Li *et al.*, 2006 ▶; Yadav *et al.*, 2005 ▶). The MPCS facilitates two styles of crystallization screening: fine-gradient screening and hybrid screening.

#### 3.1.1. Fine-gradient/optimization screening

**Figure 3:**
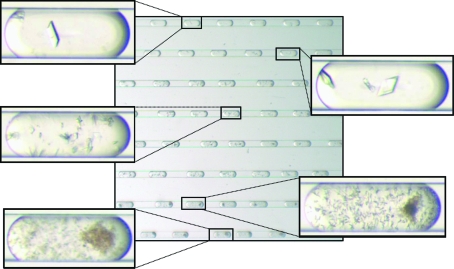
Mapping of crystallization phase space using a fine gradient for ribose-phosphate pyrophos­phokinase. A precipitant gradient demonstrates a smooth transition from precipitation (lower left) to microcrystalline precipitation (lower right) to microcrystals (middle left) to a few crystals (upper right) to single crystals (upper left) of ribose-phosphate pyrophosphokinase.

The MPCS gradient mode allows the crystallographer to very finely scan a region of crystallization phase space. Since the aqueous streams used in an MPCS experiment are independently controlled using the MPCS pumping system and the *MicroPlugger* software, concentration gradients over a series of plugs are easily formed by changing the flow rates of the individual streams (Fig. 1 ▶ *c*; Zheng *et al.*, 2003 ▶). As the precipitant stream decreases in flow rate, the buffer stream increases in flow rate such that that the sum of the flow rates remains constant (although the *MicroPlugger* software allows the user to design a customized experiment to suit their needs, including changing the plug size or the sum of the aqueous flow rates). Using a fine gradient, the crystallization phase space of a particular protein–precipitant combination can be carefully interrogated for crystal optimization or mapped out to show the transition from precipitation to microcrystals to single crystals (Fig. 3 ▶). In the same manner, so-called reverse screening can be carried out utilizing two crystallization cocktails that have been found to promote the formation of crystals (Stura *et al.*, 1994 ▶). On-chip formulation of concentration gradients can be used for the optimization of precipitants, ligands, protein partners or cryoprotectants. By reducing the volume of protein required for crystal optimization, fine-gradient formation can decrease protein-production requirements when crystals of different protein mutants or DNA complexes are required.

#### 3.1.2. Hybrid screening

Hybrid screening combines on-chip formulation of gradients with sparse-matrix screening (Li *et al.*, 2006 ▶). Sparse-matrix screening in plugs is achieved by generating a ‘pre-formed cartridge’ of plugs composed of different crystallizing agents (Zheng & Ismagilov, 2005 ▶). In the hybrid method of the MPCS, the pre-formed cartridge containing a series of ∼100 nl plugs flows into one of the three aqueous inlets of the 3+1 mixer. Using the Hybrid Mode of the *Microplugger* software, a concentration gradient of each crystallizing agent in the pre-formed cartridge is formed by coordinating the flow-rate change between the pre-formed plugs and the buffer stream. Performing sparse-matrix and gradient screening in the same trial allows the MPCS to scan a substantial portion of crystallization phase space, generating 20–40 individual crystallization experiments at different concentrations from each pre-formed crystallant plug.

## 4. Producing diffraction-ready crystals

The MPCS is more than a low-volume protein-crystallization screening tool: it produces diffraction-ready crystals. The experimental volumes (10–20 nl; the exact volumes are dependent on the CrystalCard and the flow rates) are very small compared with traditional crystallization techniques. However, 10–20 nl plugs contain enough protein to generate crystals that are large enough to obtain useful X-ray diffraction data. Crystals grown in plugs are typically 40–200 µm along their longest axis (the channel dimensions are 200 × 200 µm). Because the crystals are diffraction-ready, it is critical to have method(s) for obtaining diffraction data without scaling up the experiment or translating the crystal hit to another experimental technique. The MPCS offers two methods for obtaining X-ray diffraction data from crystals grown in plugs: traditional cryocooling by crystal extraction and *in situ* diffraction.

### 4.1. Protein-crystal extraction from the MPCS CrystalCard

**Figure 4:**
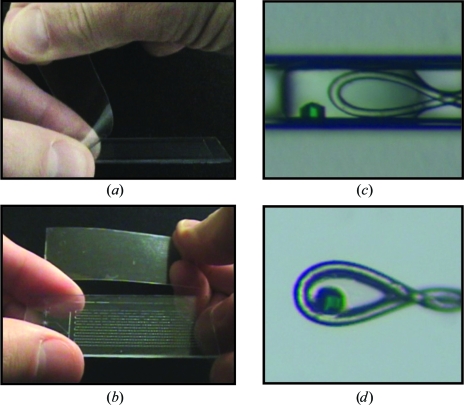
Extracting diffraction-ready crystals from the MPCS CrystalCard. (*a*) A picture of the thin plastic cover being removed from the MPCS CrystalCard. (*b*) A picture of the opened MPCS CrystalCard with all of the crystallization experiments remaining in the plastic part containing the microfluidic circuitry. (*c*) A microphotograph of a crystal in a plug just before being scooped out of the CrystalCard with a cryoloop. (*d*) A microphotograph of the crystal in the cryoloop.

Protein crystals can be extracted directly from individual plugs in the MPCS CrystalCards. A thin plastic layer is bonded to the plastic part that contains the microfluidic circuitry. This thin layer has a bond that is designed to be strong enough to prevent fluid from leaking out of the CrystalCard during the crystallization trial but weak enough to be manually peeled off (Figs. 4 ▶ *a* and 4 ▶ *b*). Additionally, the plugs are retained in the plastic part containing the microfluidic circuitry. After the thin plastic layer has been peeled back, the crystals are exposed and the desired crystal(s) are accessible to microtools such as cryoloops (Figs. 4 ▶ *c* and 4 ▶ *d*). Two techniques can be used to remove the crystals for cryoprotection: (i) the crystal(s) can be scooped out with a cryoloop and placed in the desired cryoprotectant or (ii) ∼1 µl of the desired cryoprotectant can be pipetted on top of the plugs containing crystals so that when the crystal is scooped out it is already immersed in cryo­protectant. Using the first technique, evaporation of the plugs is delayed by ∼5 min by the layer of fluorocarbon oil. Using the second technique, evaporation of the plugs is delayed for at least 20 min, providing ample time to extract many crystals.

**Figure 5:**
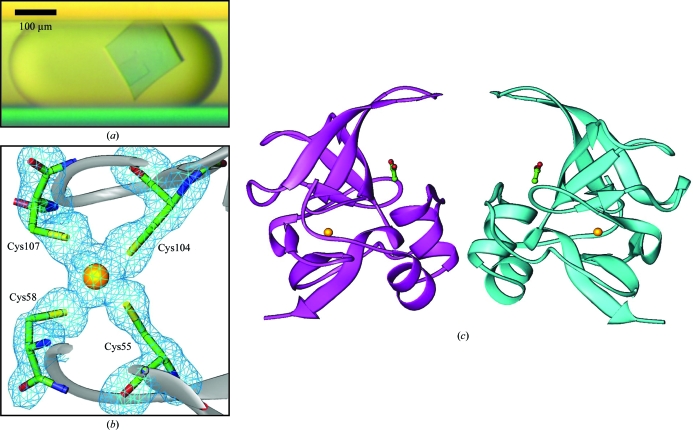
Methionine-*R*-sulfoxide reductase. (*a*) A microphotograph of a methionine-*R*-sulfoxide reductase crystal. (*b*) *F*_o_ − *F*_c_ OMIT map contoured at 3σ for Cys residues and a Zn ion. (*c*) Ribbon diagram of the NCS dimer of methionine-*R*-sulfoxide reductase from *Burkholderia pseudomallei*. Zinc ions are represented as gold spheres and acetate ions in ball-and-stick representation. The structure was deposited as PDB entry 3cxk.

Crystal extraction was used in combination with an MPCS fine-gradient screen to generate crystals of methionine-*R*-­sulfoxide reductase (Fig. 5 ▶ *a*). Crystals formed in a concentration optimization gradient screen of 30%(*v*/*v*) MPD, 25%(*w*/*v*) PEG 1500, 0.1 *M* acetate pH 4.5. The protein concentration was 22 mg ml^−1^ in 20 m*M* HEPES pH 7.0, 500 m*M* NaCl, 5% glycerol, 2 m*M* DTT. Crystals were removed from the CrystalCard using a 0.1–0.2 mm cryoloop, cryocooled (using the crystallant as the cryoprotectant) and subjected to X-ray diffraction experiments. A 1.7 Å data set was collected on SBC-CAT beamline 19BM located at the Advanced Photon Source at Argonne National Laboratories and the structure was subsequently solved and refined (Figs. 5 ▶ *b* and 5 ▶ *c*; Table 1 ▶). The final coordinates and structure factors were deposited in the Protein Data Bank (PDB code 3cxk).

### 4.2. *In situ* X-ray diffraction

**Figure 6:**
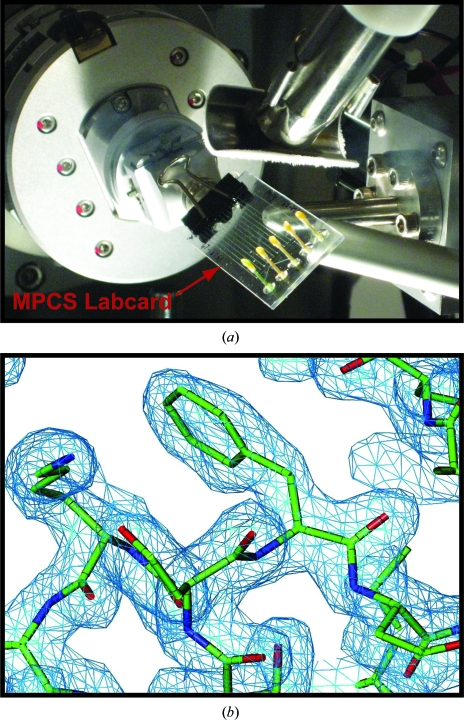
*In situ* diffraction using the MPCS. (*a*) A picture of the MPCS CrystalCard mounted on the goniometer head of an X-ray source. (*b*) A portion of the electron-density map obtained by combining *in situ* diffraction data from three crystals inside the MPCS CrystalCard.

The CrystalCard has been designed to allow *in situ* X-ray diffraction. This allows the crystallographer to assess the quality of the crystals grown before alteration by the cryoprotection process (Luft *et al.*, 1999 ▶; McPherson, 2000 ▶; Ng *et al.*, 2003 ▶; Yadav *et al.*, 2005 ▶; Lunde *et al.*, 2008 ▶) and to collect data for structure determination from robust crystals. The CrystalCard is sufficiently transparent to X-rays to be mounted directly onto a goniometer for X-ray diffraction experiments at room temperature. Additionally, the CrystalCard can be translated along its *x* and *y* axes to collect data from multiple crystals for combination to yield complete data sets. The CrystalCard can be rotated through ∼75° without hitting the beamstop. In order to demonstrate this technique, we mounted a CrystalCard containing lysozyme crystals on a goiniometer head at NE-CAT beamline 24ID-C located at the Advanced Photon Source at Argonne National Laboratories (Fig. 6 ▶ *a*) and collected X-ray diffraction data at room temperature from three crystals in the CrystalCard. A portion of the electron-density map generated by molecular replacement is shown in Fig. 6 ▶(*b*). Crystallographic data are provided in Table 1 ▶.

## 5. Materials and methods

### 5.1. Preparation of the plastic CrystalCard

Plastic CrystalCards were manufactured by injection molding (Siloam Biosciences Inc.). Plug formation in the CrystalCard requires a low surface-energy (hydrophobic) surface. This ensures that the fluorocarbon oil preferentially wets the walls of the microcapillary. The polycarbonate CrystalCard arrives from the manufacturer with a high surface-energy (hydrophilic) microcapillary surface. To pre­pare the microcapillary surface for plug formation, Cytonix PFC 502AFA solution is used to coat the inside of the microcapillary. Cytonix PFC 502AFA adheres to the polycarbonate surface and generates a coating with a surface energy of 6–10 dyn cm^−1^ (1 dyn cm^−1^ = 1 × 10^−3^ N m^−1^). To apply the coating, the CrystalCard is filled from the outlet with Cytonix 502AFA solution and incubated on ice for 2 h (the CrystalCard inlets are prone to crack if incubated at higher temperatures). The 502AFA solution is removed from the CrystalCard *via* a vacuum and clean dry air is forced through the CrystalCard at 34.5 kPa for 1 h. After drying, the CrystalCard is cured at 333 K for 1 h and is then ready for use.

### 5.2. Macro–micro interface

A macro–micro interface connection between the syringes and the CrystalCard is required for the MPCS to function properly. A 360 µm inner diameter, 760 µm outer diameter (ID/OD 360/760) section of Teflon tubing is used to connect the syringe to the CrystalCard. One end of the Teflon tubing slides onto the syringe needle and the other end slides into a 760 µm inner diameter piece of silicon tubing connected to the plastic CrystalCard. For the PDMS/Teflon CrystalCard, holes are manufactured in the PDMS to allow a tight-fitting seal with the ID/OD 360/760 Teflon tubing.

### 5.3. Preparation of the hybrid method pre-formed cartridge (Li *et al.*, 2006 ▶)

A 5–10 cm piece of ID/OD 360/760 Teflon tubing was connected to a 50 µl syringe and the other end to a 10 cm piece of 200 µm inner diameter/230 µm outer diameter (ID/OD 200/230) Teflon storage capillary. The connection was sealed with capillary wax (Hampton Research). The syringe and tubings were filled with FC40 (3M) and the syringe was mounted on a manual aspirator (Stoelting No. 262M=, Wood Dale, Illinois, USA). The end of the ID/OD 200/230 Teflon storage capillary was dipped into the first precipitant and ∼150 nl of fluid (15 units on the aspirator) was aspirated. A ∼50 nl air bubble (5 units on the aspirator) was aspirated. This sequence was repeated until all of the desired precipitants (up to 24) had been aspirated. 2 µl FC40 (∼200 units on the aspirator) were aspirated into the tubing and the syringe was placed on the pump for the experiment. The end of the ID/OD 200/230 Teflon storage capillary containing the pre-formed cartridge was connected to the precipitant inlet of the PDMS/Teflon CrystalCard by cutting the channel with a razor blade and inserting the capillary horizontally. The end of the ID/OD 200/230 Teflon storage capillary was inserted into the PDMS chip (up to the point in the inlet where it becomes tapered) and sealed using capillary wax.

### 5.4. Structure determination

Data sets were collected at the Advanced Photon Source: beamline 19BM at 100 K for methionine-*R*-sulfoxide reductase and beamline 24-IDC at room temperature for lysozyme. Data were integrated and scaled with *HKL*-2000 (Otwinowski & Minor, 1997 ▶). For the lysozyme structure, intensities were integrated separately for each of the three data sets using the *MOSFLM* package (Leslie, 1992 ▶). The structures of lysozyme and methionine-*R*-sulfoxide reductase were solved by molecular replacement using *MOLREP* (Vagin & Teplyakov, 1997 ▶) with PDB entries 1iee and 3cez as the search models, respectively. Structures were refined with *REFMAC*5 (Murshudov *et al.*, 1997 ▶) and model building was performed with *Coot* (Emsley & Cowtan, 2004 ▶).

### 5.5. X-ray absorption of the CrystalCard

A simple test was conducted to analyze the absorption of X-­rays by the CrystalCard. To do this, the beam current in the ion chamber normalized to the APS ring current (*I*/*I*_0_) was measured with and without the CrystalCard inserted at a wavelength of 0.979261 Å (12.66099 keV). *I*/*I*_0_ without the CrystalCard was 1.91671 × 10^−6^ and *I*/*I*_0_ with the CrystalCard (perpendicular to the beam) was 1.5511 × 10^−6^. This constitutes a 19% X-ray absorbance by the CrystalCard.

## Supplementary Material

PDB reference: methionine-*R*-sulfoxide reductase, 3cxk, r3cxksf

## Figures and Tables

**Table 1:** Crystallographic data Values in parentheses are for the 2.00–1.90 Å resolution shell for lysozyme and the 1.76–1.70 Å shell for methionine-*R*-sulfoxide reductase.

	Lysozyme	Methionine-*R*-sulfoxide reductase
Data collection		
Unit-cell parameters (Å)	*a* = *b* = 79.18, *c* = 38.38, α = β = γ = 90	*a* = 42.00, *b* = 45.17, *c* = 45.40, α = 88.4, β = 83.7, γ = 69.1
Space group	*P*4_3_2_1_2 (No. 96)	*P*1 (No. 1)
Resolution (Å)	50–1.90	50–1.70
Wavelength (Å)	0.97950	0.97932
Total reflections	54338	118181
Unique reflections	10151	32539
〈*I*/σ(*I*)〉	11.4 (2.9)	23.1 (2.2)
*R*_merge_† (%)	13.7 (58.4)	6.8 (42.3)
Completeness (%)	98.8 (98.5)	95.3 (87.4)
Redundancy	5.4 (5.0)	3.6 (3.3)
Wilson *B* factor (Å^2^)	24.1	22.1
Refinement		
Resolution (Å)	50–1.90	50–1.70
Reflections (working/test)	9412/480	30828/1650
*R*_working_/*R*_free_‡ (%)	19.6/23.0	16.6/19.9
No. of atoms (protein/solvent)	1001/45	2082/180
R.m.s. deviations		
Bond lengths (Å)	0.016	0.015
Bond angles (°)	1.607	1.408
Average *B* factor (Å^2^)		
All atoms	28.8	28.3
Protein	28.5	27.5
Water	35.9	37.0
Coordinate error based on *R*_free_ (Å)	0.149	0.095
Ramachandran analysis (%)		
Most favored (chain *A*/*B*)	89.4	91.7/90.8
Additionally allowed (chain *A*/*B*)	10.6	7.3/8.3

† *R*_merge_ = 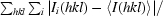 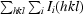, where *I*_*i*_(*hkl*) is the intensity measured for the *i*th reflection and 〈*I*(*hkl*)〉 is the average intensity of all reflections with indices *hkl*.

‡ *R*_working_ = 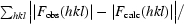 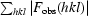; *R*_free_ is calculated in an identical manner using 5% of randomly selected reflections that were not included in the refinement.
